# Talampanel reduces the level of motoneuronal calcium in transgenic mutant SOD1 mice only if applied presymptomatically

**DOI:** 10.3109/17482968.2011.584627

**Published:** 2011-05-30

**Authors:** Melinda Paizs, Massimo Tortarolo, Caterina Bendotti, Jozsef I Engelhardt, László Siklós

**Affiliations:** 1 Laboratory of Molecular Neurobiology, Institute of Biophysics, Biological Research Centre, Szeged, Hungary; 2 Laboratory of Molecular Neurobiology, Department of Neuroscience, ‘Mario Negri’ Institute for Pharmacological Research, Milan, Italy; 3 Department of Neurology, University of Szeged, Hungary

**Keywords:** Talampanel, AMPA receptor, mSODl model, intracellular calcium, motor neuron

## Abstract

We tested the efficacy of treatment with talampanel in a mutant SOD1 mouse model of ALS by measuring intracellular calcium levels and loss of spinal motor neurons. We intended to mimic the clinical study; hence, treatment was started when the clinical symptoms were already present. The data were compared with the results of similar treatment started at a presymptomatic stage. Transgenic and wild-type mice were treated either with talampanel or with vehicle, starting in pre-symptomatic or symptomatic stages. The density of motor neurons was determined by the physical disector, and their intracellular calcium level was assayed electron microscopically. Results showed that motor neurons in the SOD1 mice exhibited an elevated calcium level, which could be reduced, but not restored, with talampanel only when the treatment was started presymptomatically. Treatment in either presymptomatic or symptomatic stages failed to rescue the motor neurons. We conclude that talampanel reduces motoneuronal calcium in a mouse model of ALS, but its efficacy declines as the disease progresses, suggesting that medication initiation in the earlier stages of the disease might be more effective.

## Introduction

Amyotrophic lateral sclerosis (ALS) is a multifactorial (1), multisystem (2), non-cell autonomous disease (3). Several pathomechanisms of the degeneration of motor neurons (MNs) have been identified, which contribute to the progression of the motor dysfunction during the disease. Excitotoxicity has attracted considerable attention since an abnormal glutamate metabolism was documented in pathogenesis-oriented clinical studies (4), and was confirmed in in vitro experiments and in animal models of ALS (5). Moreover, riluzole, the only drug to date to display a potential to increase patient survival, has an anti-excitotoxic effect (6). Since glutamate-induced excitotoxicity to MNs is mediated through calcium-permeable alpha-amino-3-hydroxy-5-methyl-4-isoxazolepropionic acid (AMPA) receptors (7), a protective effect was expected from treatment with AMPA receptor antagonists. Indeed, different competitive (8) or non-competitive (9,10) AMPA receptor inhibitors have been reported to prolong the survival of transgenic (Tg) mice with mutant Cu/Zn superoxide dismutase (SOD1) when administered before the onset of motor impairment. Using the non-competitive AMPA receptor inhibitor, we modestly reduced the loss of MNs in the lumbar spinal cord of SOD1-G93A mice, while the morphology of the remaining MNs, and especially that of the den-drites was well preserved by the treatment (9). As AMPA receptor-mediated excitotoxicity on MNs in in vitro paradigms is thought to be mediated by an excessive calcium influx particularly at the level of the dendrites, we hypothesized that the beneficial effect of AMPA receptor inhibitors could be a result of a reduced permeability to calcium. The drug used in our previous study belongs in the class of active non-competitive antagonists (or negative allosteric modulators) of the AMPA subtype of glutamatergic excitatory amino acid receptor inhibitors similar to talampanel (8-methyl-7H-1,3-dioxolo(2,3)benzodiazepine) (10).

Talampanel, an orally active drug, was recently selected for a clinical trial (11), which unfortunately did not meet the expected efficacy in reducing the disease-related functional deterioration in ALS patients. In an attempt to resolve the mismatch between the results of the animal studies and the clinical trial we tested the efficacy of talampanel treatment by direct assay of the intracellular calcium level and the number of surviving spinal MNs in mutant SOD1 Tg mice in presymptomatic and symptomatic age groups.

## Methods

### Animals

Hemizygous Tg mice, expressing mutant human SOD1 with a G93A substitution, obtained originally from Jackson Laboratories, were bred and maintained in a C57BL/ 6JOlaHsd mice strain at the ‘Mario Negri’ Institute for Pharmacological Research, Milan, Italy. The animals were housed at a temperature of 21°C under a 12-h dark/light cycle. Food (standard pellets) and water were supplied ad libitum. Female mutant SOD1 Tg mice were treated orally with talampanel (5 mg/kg body weight dissolved in 0.1 mlTween 80) or with vehicle only once a day for two weeks. The oral 5 mg/kg dose of talampanel was selected on the basis of no observed adverse effect on body weight gain (TEVA, personal communication) and on a pilot study in our laboratory showing that this dose was effective in ameliorating mitochondrial functionality in mutant SOD1 Tg mice (unpublished data). Animals were treated at the same hour in the morning every day, without anaesthesia, by direct administration into the lower oesophagus by using an appropriate stainless steel gavage needle with a round tip. Treatments were started at 10 or 17 weeks of age, i.e. ages corresponding to the presymptomatic stage and to the onset of the motor dysfunction. Age-matched female non-Tg mice that were treated similarly were used as controls. Procedures involving animals and their care were conducted in full conformity with the institutional guidelines, which are in compliance with national (D.L. No. 116, G.U. Supplement 40, February 18, 1992, Circolare No. 8, G.U., 14 luglio 1994) and international (EEC Council Directive 86/ 609, OJ L 358, 1 DEC. 12, 1987; NIH Guide for the Care and Use of Laboratory Animals, United States National Research Council, 1996) laws and policies.

### Specimen preparation

Under terminal anaesthesia with Equitensin (1% phenobarbital, 4% (v/v) chloral hydrate; 6 μl/g, intraperitoneally), mice in the two age groups (at 12 weeks or 19 weeks of age; *n* = 12 in each group) were perfused transcardially with a glutaraldehyde fixative containing potassium oxalate for simultaneous preservation of the ultrastructure and precipitation of the tissue calcium, described in detail earlier (12). Transverse slices 2-3 mm in thickness were removed from lumbar segments of the spinal cords and processed by the oxalate-pyroantimonate method for visualization of the tissue calcium electron microscopically as electron-dense deposits (EDDs) (12). From the same plastic-embedded tissue blocks, series of ultra-thin (thickness 50 nm) and semi-thin (thickness 300 nm) sections were prepared to determine the volume density of the EDDs in the lumbar MNs under the electron microscope, and to assay the numerical density of these cells light microscopically.

### Quantification of the calcium level and the volume density of MNs

The level of calcium in the perikaryonal region of the MNs was considered to be reflected by the volume density of the EDDs and was determined by point-counting methods in electron microscopic prints. The technique was adapted to neural tissue in an earlier study (13). Pooled data were expressed as percentages of the perikaryonal volume occupied by the EDDs. An unbiased estimation of the number of MNs in the lumbar segment of the spinal cord was performed by using the physical disector, optimized for sampling such cells in our earlier experiments (14). Pairs of 300-nm-thick toluidine blue-stained sections, cut at a distance of 8 μm, were used for the study. Data were expressed as cell numbers in mm^3^ of spinal cord tissue for each animal.

The cell numbers and the relative volumes of the EDDs in the treatment groups were expressed as means ± SEM. The effects of treatment in the two strains were evaluated by two-way ANOVA with Duncan post hoc comparison for the two age groups.

## Results

**Figure 1:**
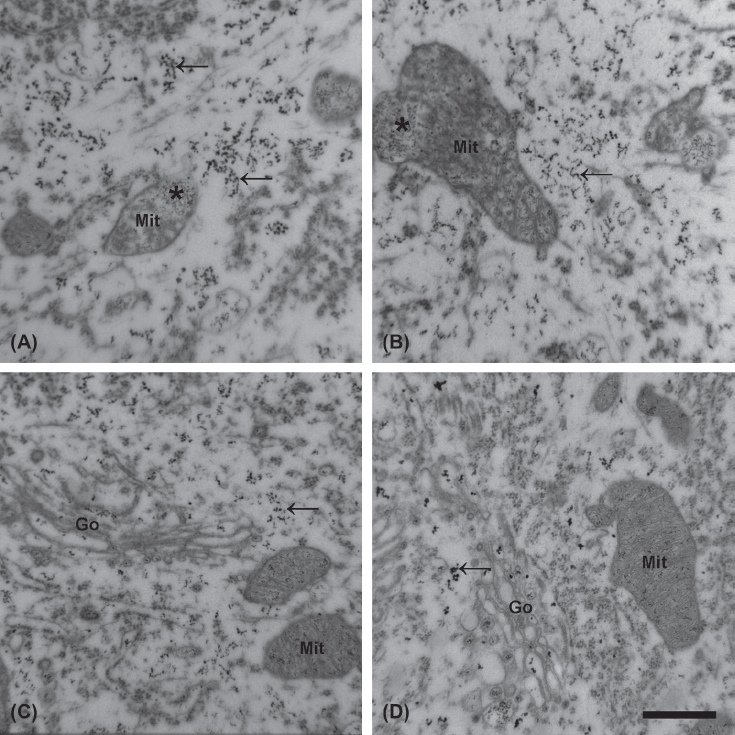
Increased calcium level in the lumbar motor neurons of mutant SOD1 Tg mice. An increased number of electron-dense deposits (EDDs; arrows) is noted, reflecting the distribution of calcium in the motor neurons in the talampanel-treated (A) and the vehicle-treated (B) mutant SOD1 Tg mice at the age of 19 weeks. Motor neurons from the wild-type mice at the same age contain fewer deposits, regardless of whether they were treated with talampanel (C) or with vehicle only (D). Besides the increased number of EDDs in the mutant SOD1 Tg animals, mitochondrial degeneration with clusters of EDDs (asterisk) was frequently seen. In the wild-type animals, regardless of the treatment, no structural alterations were present. Mit: mitochondrion, Go: Golgi complex. Bar: 500 nm.

**Figure 2:**
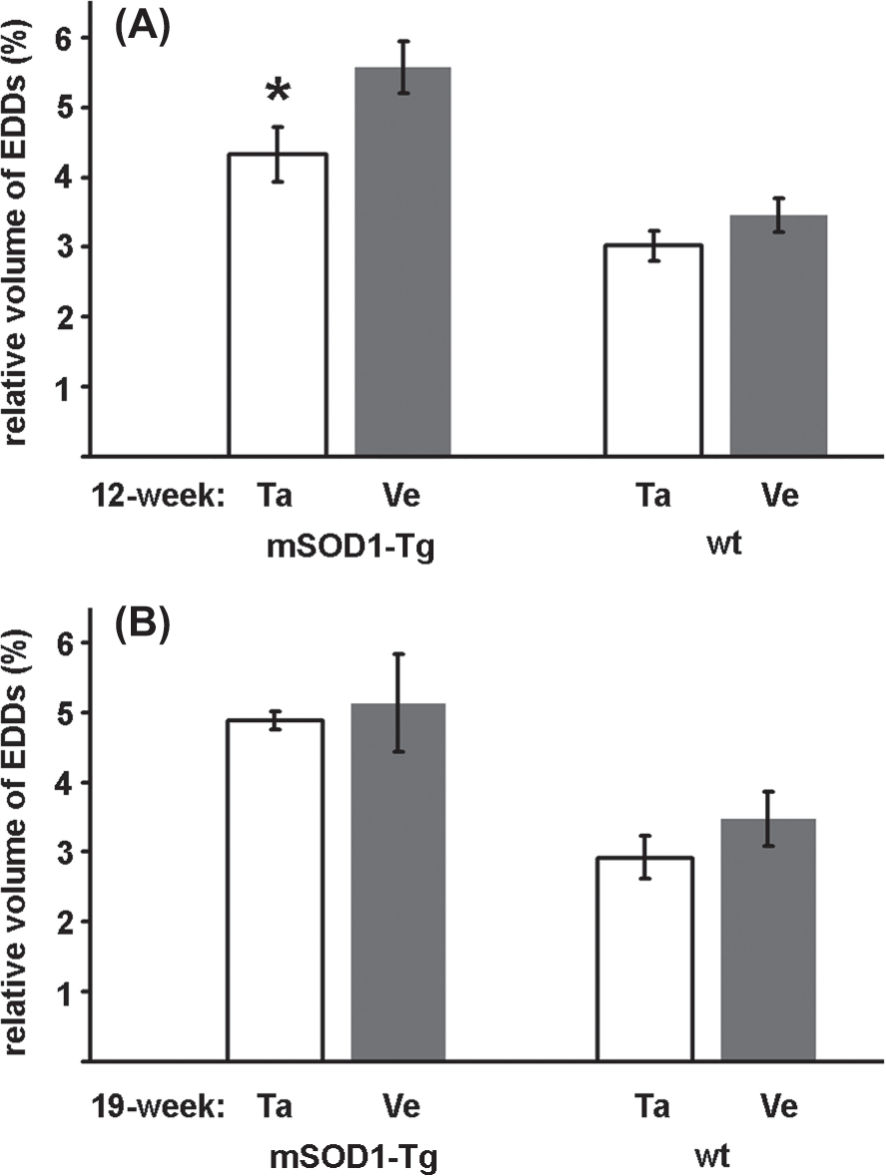
Effects of talampanel treatment on the calcium level of lumbar motor neurons of mutant SOD1 Tg mice. The volume occupied by electron-dense deposits (EDDs), reflecting tissue calcium, relative to the perikaryal volume was determined in mutant SOD1 Tg (mSODl-Tg) and wild-type (wt) animals after talampanel (Ta) or vehicle (Ve) treatment at 12 (A) and 19 (B) weeks of age. Talampanel had a significant effect in reducing the calcium level only at the age of 12 weeks (asterisk; *p* = 0.01, two-way ANOVA with Duncan post hoc comparison). Data are shown as means ± SEM.

In parallel with the morphological signs of degeneration, a higher number of EDDs, reflecting an increased level of calcium, was seen in the cytoplasm of the surviving MNs of the mutant SOD 1 Tg animals compared with wild-type ones (Figure 1). The treatment with talampanel reduced the elevated calcium level to a significant extent, but only in the group aged 12 weeks (Figure 2A, B). At this age, a 62% increase (5.57 ±0.37% vs. 3.45 ±0.24%; Figure 2A) in the volume density of the EDDs was noted in the vehicle-treated Tg animals relative to the similarly treated wild-type mice (*p* = 0.0002). This elevated calcium level was reduced by a significant 22% by talampanel treatment (to 4.33 ± 0.39%; *p* = 0.01) (Figure 2A), although this level was still significantly higher than that for the wild-type group (3.02 ± 0.21%; *p* = 0.009) (Figure 2A). At 19 weeks of age, the MNs from the vehicle-treated mutant SOD1 Tg mice displayed similar increases in the volume density of the EDDs to those from the 12-week-old animals relative to the wild-type mice (5.13 ± 0.70% vs. 3.15 ± 0.39%; *p* = 0.014) (Figure 2B), but a comparison with the corresponding talampanel-treated mutant SOD1 Tg mouse group demonstrated that talampanel was non-effective (4.88 ±0.13%; *p* = 0.721) (Figure 2B).

**Figure 3:**
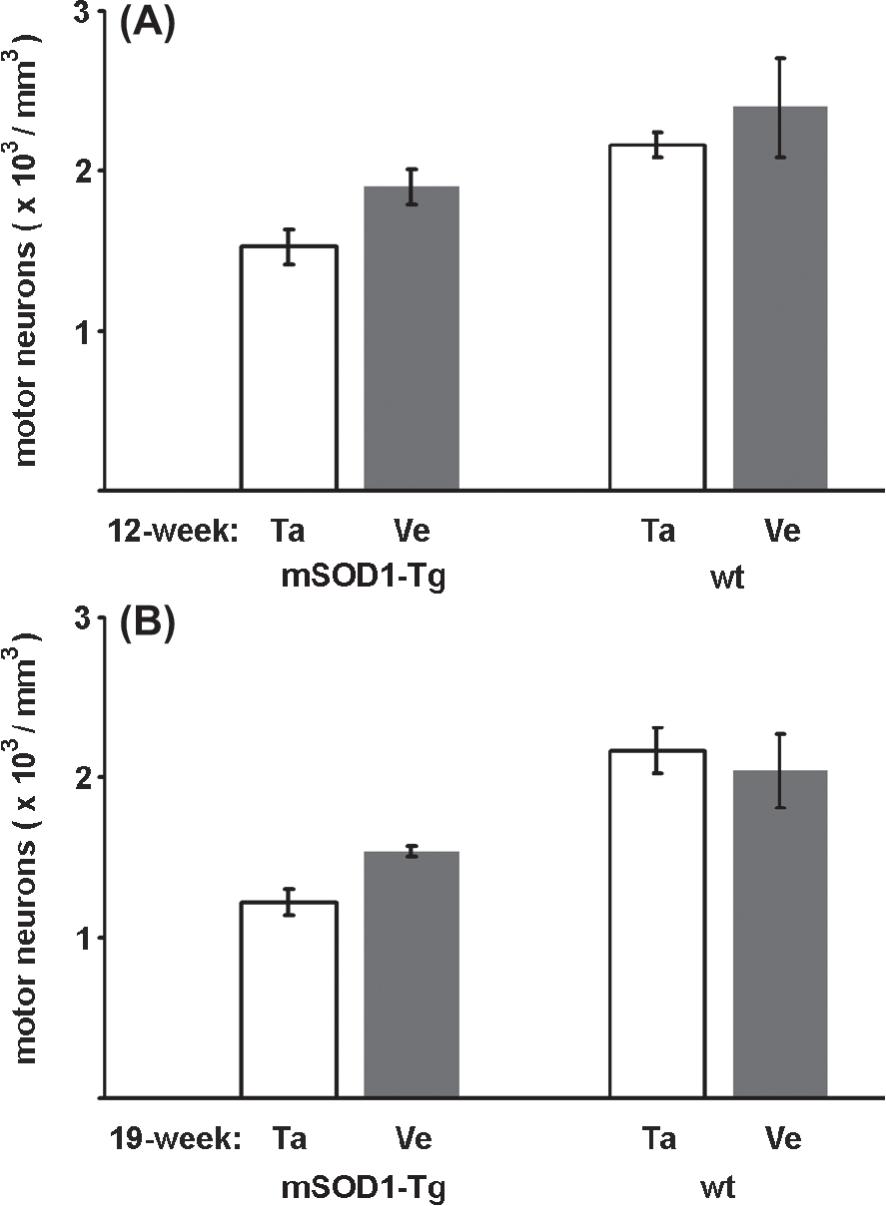
Effects of talampanel treatment on the number of lumbar motor neurons in mutant SOD1 Tg mice. Relative to the vehicle-only treatment (Ve), talampanel treatment (Ta) did not exert a significant effect either in the mutant SOD1 Tg (mSOD 1-Tg), or in the wild-type (wt) mice in the presymptomatic (A) or a later stage of the disease (B). Regardless of the treatment, lower cell numbers were counted at 19 weeks of age than at 12 weeks of age in the mutant SOD1 Tg animals. Data are shown as means ± SEM.

With regard to the number of surviving MNs at the age of 12 weeks, the difference between the talampanel- and vehicle-treated groups was not significant either in the mutant SOD1 Tg group (1.524 ±0.104 and 1.899 ± 0.107; *p* = 0.183) (Figure 3A), or in the wild-type group (2.161 ± 0.078 and 2.396 ±0.310; *p* = 0.398) (Figure 3A). At the age of 19 weeks, the differences in the mutant SOD1 Tg group (1.222 ±0.083 and 1.537 ±0.037; *p* = 0.093) (Figure 2B) and the wild-type group (2.166 ±0.142 and 2.041 ±0.229; *p* = 0.488) (Figure 2B) were likewise not significant.

Disregarding the non-significant effect of talampanel on the number of MNs, the mutant SOD1 Tg and wild-type mouse strains were compared directly in both age groups. This comparison revealed an overall cell loss of 25% at 12 weeks of age in the mutant SOD1 Tg animals relative to the wild-type controls, before any motor deficit was detectable, and the MN loss rose to 35% at 19 weeks of age (*p* = 0.005 and *p* = 0.0001, respectively; one-way AN OVA with Duncan post hoc comparison).

## Discussion

Since no significant effect of talampanel on the number of MNs could be demonstrated in either of the mouse strains in the present study, we pooled the numerical density data for the mutant SOD1 Tg and wild-type strains in the two age groups. The comparison with non-Tg littermates revealed an overall cell loss of 25% at 12 weeks of age in the mutant SOD1 Tg animals, before any motor deficit was detectable. At 19 weeks of age, the mutant SOD1 Tg mice exhibited a decline of about 50% in their grip strength (15), which may be compared with a 35% cell loss obtained in our experiments, since even at a late symptomatic stage the reported loss of MNs does not exceed 50% (16). Otherwise, our finding that talampanel itself had no effect on the number of spinal MNs in the wild-type mice suggests that the drug (at the applied dose) may safely be used in these animals.

In accordance with the first description of an altered calcium level in the MNs of mutant SOD1 Tg animals (17), a higher calcium level was detected in the surviving MNs compared with the wild-type mice. Our empirical approach to quantify the relative amount of EDDs as proportional to the concentration of intracellular calcium is based on the cytochemical precipitation of calcium as EDDs, and visualization of the EDDs under the electron microscope. Comparable techniques in the literature, based on calcium precipitation with oxalate-pyroantimonate (18,19),bichromate (20),orphosphate-chromium(III)-trisoxalate (21), probably reveal a fraction of the mobile, or loosely bound calcium (21). In this study, a glutaraldehyde fixative containing oxalate anions was employed to conserve the distribution of calcium and preserve the structure for electron microscopy (18,19). The method for the quantification of the distribution of calcium, based on identification of the pattern of EDDs with that of calcium, was confirmed in earlier experiments, and proved useful for following slowly-evolving function-dependent changes in the intracellular calcium in a variety of experimental paradigms (22-26), including the first description of an increased calcium level in the motor nerve terminals in muscle biopsies from ALS patients (27) and in the MNs of mutant SOD1 Tg animals (17); it was also suitable in the present study for an assessment of the effect of talampanel on the calcium level of the MNs.

The demonstrated effect of talampanel in reducing motoneuronal calcium if applied presymptomatically suggests that the rationale of the treatment is basically correct. However, the lack of any effect at the symptomatic stage indicates that the drug loses its efficacy as the disease progresses. Nonetheless, even though talampanel was effective in reducing the level of intracellular calcium in the presymptomatic stage, no effect was seen on the rescue of surviving MNs, and this may provide a hint that talampanel actually exerts its expected effect, but is overwhelmed by the increasing pathogenic mechanisms. Thus, the ultimate failure of treatment with talampanel may possibly be attributed, at least in part, to the late initiation of the medication relative to the disease progression and/or to the lack of supplementation with other drugs compensating additional components of the pathomechanism. These results underscore the importance of estimation of the efficacy of drug action in both the presymptomatic and symptomatic phases during preclinical animal experiments (28), and accentuate the need for early diagnostic biomarkers for ALS which may facilitate clinical trials and will presumably allow earlier initiated and more efficacious therapy (29).
